# Supplementary material for: COVID-19–related perceptions, context and attitudes of adults with chronic conditions: Results from a cross-sectional survey nested in the ComPaRe e-cohort
Source: PLoS One. 2020 Aug 6;15(8):e0237296. doi: 10.1371/journal.pone.0237296 (PMC7410193; doi:10.1371/journal.pone.0237296)
Supplement: S1 Data — (DOCX) [file pone.0237296.s001.docx]

**S1 Data. Questionnaire for participants (French)**

**Introduction**

Comme vous le savez, la France est actuellement touchée par l’épidémie de coronavirus Covid-19. Depuis le début de l’épidémie, les patients souffrant de maladies chroniques sont considérés comme à risque d’une forme d’infection sévère. A ce jour, les recommandations concernant les patients à risque sont limitées et la conduite à tenir incertaine. A l’aide de ComPaRe, nous aimerions étudier la perception, par les patients chroniques, de l’épidémie, et de votre attitude, en tant que patients avec des maladies chroniques. Ces données seront cruciales pour aider à mieux communiquer avec les patients souffrant de maladies chroniques. Afin que l’analyse soit la plus pertinente possible, n’oubliez pas de mettre à jour votre dossier de santé (lien ici) et de compléter vos autres questionnaires ComPaRe. Nous avons besoin de votre aide pour lutter contre cette épidémie. Merci de votre aide !

**Votre santé, aujourd'hui**

Les questions suivantes n'ont pas pour objectif de juger si ce que font les patients est bien ou pas, mais d’évaluer le risque d’infection.

Avez-vous consulté un soignant (généraliste, spécialiste, kinésithérapeute...) dans les 30 derniers jours ?

- 1 - Oui
- 2 - Non

Au cours de ces 30 derniers jours, avez-vous du vous rendre physiquement dans un lieu de soins (cabinet médical, hôpital, etc.) ?

- 1 - Oui
- 2 - Non

Décrivez les mesures de protection que vous avez pris lors de ces occasions ?

- 1 - Aucune mesure particulière
- 2 - Distance d'un mètre minimum avec toute autre personne
- 3 - Port d'un masque
- 4 - Port de gants
- 5 - Autre

Au cours de ces 30 derniers jours, avez-vous du vous rendre physiquement dans une pharmacie ?

- 1 - Oui
- 2 - Non

Décrivez les mesures de protection que vous avez pris à la pharmacie ?

- 1 - Aucune mesure particulière
- 2 - Distance d'un mètre minimum avec toute autre personne
- 3 - Port d'un masque
- 4 - Port de gants
- 5 - Autre

**Perception du risque**

Les questions suivantes n'ont pas pour objectif de juger si ce que font les patients est bien ou pas, mais d’évaluer le risque d’infection.

Vous sentez vous à risque accru d’une forme d’infection sévère par le coronavirus par rapport à des personnes du même âge que vous, mais sans maladies chronique ? Il n’y a pas de bonne ou de mauvaise réponse. Aujourd’hui, les catégories de personne à risque restent très simples et ne couvrant pas l’immense diversité des maladies et traitements possibles (liste des personnes à risque).

- 1 - Oui (à cause de mes maladies ou de mes traitements)
- 2 - Non

**Au domicile**

Les questions suivantes n'ont pas pour objectif de juger si ce que font les patients est bien ou pas, mais d’évaluer le risque d’infection.

Actuellement, continuez-vous à travailler en dehors de chez vous ?

- 1 - Oui
- 2 - Non (télétravail, chômage partiel)

Etes-vous un professionnel de santé (avec une activité clinique) ?

- 1 - Oui
- 2 - Non

Combien de personnes vivent à votre domicile (y compris vous-même) ?

|  |
| --- |

D’autres personnes vivant avec vous à votre domicile travaillent-elles en dehors de chez vous ?

- 1 - Oui
- 2 - Non (télétravail, chômage partiel)

Parmi ces personnes, certaines sont-elles des professionnels de santé (avec une activité clinique) ?

- 1 - Oui
- 2 - Non

Parmi ces personnes, certaines personnes sont-elles en contact fréquent avec le grand public ?

- 1 - Oui
- 2 - Non

Avez-vous des enfants

- 1 - Oui
- 2 - Non

Ces enfants sont-ils gardés à domicile ?

- 1 - Oui
- 2 - Non

**Contacts extérieurs fréquents**

Les questions suivantes n'ont pas pour objectif de juger si ce que font les patients est bien ou pas, mais d’évaluer le risque d’infection.

Depuis le début de l’épidémie, continuez-vous à recevoir régulièrement des personnes extérieures à votre domicile (famille, femme de ménage, garde d’enfant, amis, etc.) ?

- 1 - Oui
- 2 - Non

Décrivez les mesures de protection que vous prenez/prendriez avec ces personnes (contacts fréquents) si elles n’ont PAS de symptôme ?

- 1 - Distance d'un mètre minimum avec toute autre personne
- 2 - Port d'un masque
- 3 - Port de gants
- 4 - Autre

Quelle est/serait votre attitude si ces personnes ONT des symptômes ?

- 1 - Distance d'un mètre minimum avec toute autre personne
- 2 - Port d'un masque
- 3 - Port de gants
- 4 - Autre

**Contacts extérieurs occasionnels**

Les questions suivantes n'ont pas pour objectif de juger si ce que font les patients est bien ou pas, mais d’évaluer le risque d’infection.

Depuis le début de l’épidémie, décrivez les mesures de protection que vous prenez en cas de contact occasionnel avec des personnes qui n’ont PAS de symptômes (courses, rencontre avec des voisins, aller chercher son courrier) ?

- 1 - Distance d'un mètre minimum avec toute autre personne
- 2 - Port d'un masque
- 3 - Port de gants
- 4 - Autre

Quelle est/serait votre attitude si ces personnes ONT des symptômes ?

- 1 - Distance d'un mètre minimum avec toute autre personne
- 2 - Port d'un masque
- 3 - Port de gants
- 4 - Autre

**Isolement au domicile**

Les questions suivantes n'ont pas pour objectif de juger si ce que font les patients est bien ou pas, mais d’évaluer le risque d’infection.

Si vous ou une autre personne vivant à votre domicile deviez-vous isoler (par exemple, en cas d’apparition d’une fièvre et d’une toux inhabituelle), disposez-vous :

- 1 - D’une chambre séparée des autres occupants du domicile
- 2 - D’une salle de bain séparée des autres occupants du domicile
- 3 - De masques chirurgicaux (environ 30 masques)
- 4 - Aucun des éléments au-dessus

**Commentaires**

Avez-vous des commentaires à apporter à vos réponses ? (Merci de ne pas mettre votre nom ou toute information permettant votre identification dans cet espace. Si vous rencontrez des difficultés pour compléter ce questionnaire, contactez notre équipe via la rubrique Contact de votre espace personnel)
