# Supplementary material for: COVID-19–related perceptions, context and attitudes of adults with chronic conditions: Results from a cross-sectional survey nested in the ComPaRe e-cohort
Source: PLoS One. 2020 Aug 6;15(8):e0237296. doi: 10.1371/journal.pone.0237296 (PMC7410193; doi:10.1371/journal.pone.0237296)
Supplement: S2 Data — (DOCX) [file pone.0237296.s002.docx]

# **S2 Data. Questionnaire for participants (translated)**

**Introduction**

As you know, France is currently affected by the Covid-19 epidemic. Since the beginning of the epidemic, patients with chronic conditions have been considered at risk of severe illness. To date, recommendations concerning patients at risk are limited. With the help of ComPaRe, we would like to study how chronic patients perceive the epidemic and your attitude as patients with chronic diseases. This data will be crucial in helping to better communicate with patients with chronic diseases.

We need your help to fight this epidemic.

Thank you for your help!

**Visits to care structures**

Have you consulted a care provider (general practitioner, specialist, physiotherapist...) in the last 30 days?

○ 1 - Yes

○ 2 – No

In the last 30 days, did you visit (physically) a care structure (doctor's office, hospital, etc.)?

○ 1 - Yes

○ 2 – No

Describe the protective measures you took on these occasions?

□ 1 - No special measures

□ 2 - Distance of at least one meter with any other person

□ 3 - Wearing a mask

□ 4 - Wearing gloves

□ 5 – Other

In the last 30 days, have you had to go to a pharmacy?

○ 1 - Yes

○ 2 – No

Describe the protective measures you took at the pharmacy?

□ 1 - No special measures

□ 2 - Distance of at least one meter with any other person

□ 3 - Wearing a mask

□ 4 - Wearing gloves

□ 5 - Other

**Perception of risk**

Do you feel at increased risk of a severe form of coronavirus infection compared to people of the same age as you but without chronic disease? There is no right or wrong answer.

○ 1 - Yes (because of my illnesses or treatments)

○ 2 - No

**At home**

The following questions are not intended to judge whether what patients do is good or bad, but to assess the risk of infection.

Do you currently continue to work outside the home?

○ 1 - Yes

○ 2 - No (teleworking, short-time working)

Are you a health professional (with a clinical activity)?

○ 1 - Yes

○ 2 – No

How many people live in your home (including yourself)?

|  |
| --- |

Do other people living with you at your home work outside your home?

○ 1 - Yes

○ 2 - No (teleworking, short-time working)

Among these people, are some of them health professionals (with a clinical activity)?

○ 1 - Yes

○ 2 – No

Are any of these people in frequent contact with the general public?

○ 1 - Yes

○ 2 – No

Do you have children

○ 1 - Yes

○ 2 – No

Are these children being kept at home?

○ 1 - Yes

○ 2 - No

**Frequent external contacts**

The following questions are not intended to judge whether what patients do is good or bad, but to assess the risk of infection.

Since the beginning of the epidemic, have you continued to receive regular visits from people outside your home (family, housekeeper, childcare provider, friends, etc.)?

○ 1 - Yes

○ 2 – No

Describe the protective measures you take/will take with these people (frequent contact) if they have NO symptoms?

□ 1 - Distance of at least one meter with any other person

□ 2 - Wearing a mask

□ 3 - Wearing gloves

□ 4 – Other

What is/would be your attitude if these people HAVE symptoms?

□ 1 - Distance of at least one meter with any other person

□ 2 - Wearing a mask

□ 3 - Wearing gloves

□ 4 - Other

**Occasional external contacts**

The following questions are not intended to judge whether what patients do is good or bad, but to assess the risk of infection.

Since the beginning of the epidemic, describe the protective measures you take in case of occasional contact with people who have NO symptoms (shopping, meeting neighbors, picking up mail)?

□ 1 - Distance of at least one meter with any other person

□ 2 - Wearing a mask

□ 3 - Wearing gloves

□ 4 – Other

What is/would be your attitude if these people HAVE symptoms?

□ 1 - Distance of at least one meter with any other person

□ 2 - Wearing a mask

□ 3 - Wearing gloves

□ 4 – Other

**Isolation at home**

The following questions are not intended to judge whether what patients do is good or bad, but to assess the risk of infection.

If you or another person living in your home need to isolate (for example, if you develop a fever and an unusual cough), please be prepared to:

□ 1 - A room separate from the other occupants of the home

□ 2 - A bathroom separated from the other occupants of the home

□ 3 - Surgical masks (about 30 masks)

□ 4 - None of the above

**Comments**

Do you have any comments on your answers? (Please do not put your name or any identifying information in this space. If you encounter any difficulties in completing this questionnaire, please contact our team via the Contact section of your personal space)
