# Supplementary figures and images for: COVID-19–related perceptions, context and attitudes of adults with chronic conditions: Results from a cross-sectional survey nested in the ComPaRe e-cohort
Source: PLoS One. 2020 Aug 6;15(8):e0237296. doi: 10.1371/journal.pone.0237296 (PMC7410193; doi:10.1371/journal.pone.0237296)

**S1 Fig. Flow chart of participants’ answers to the survey**.

**
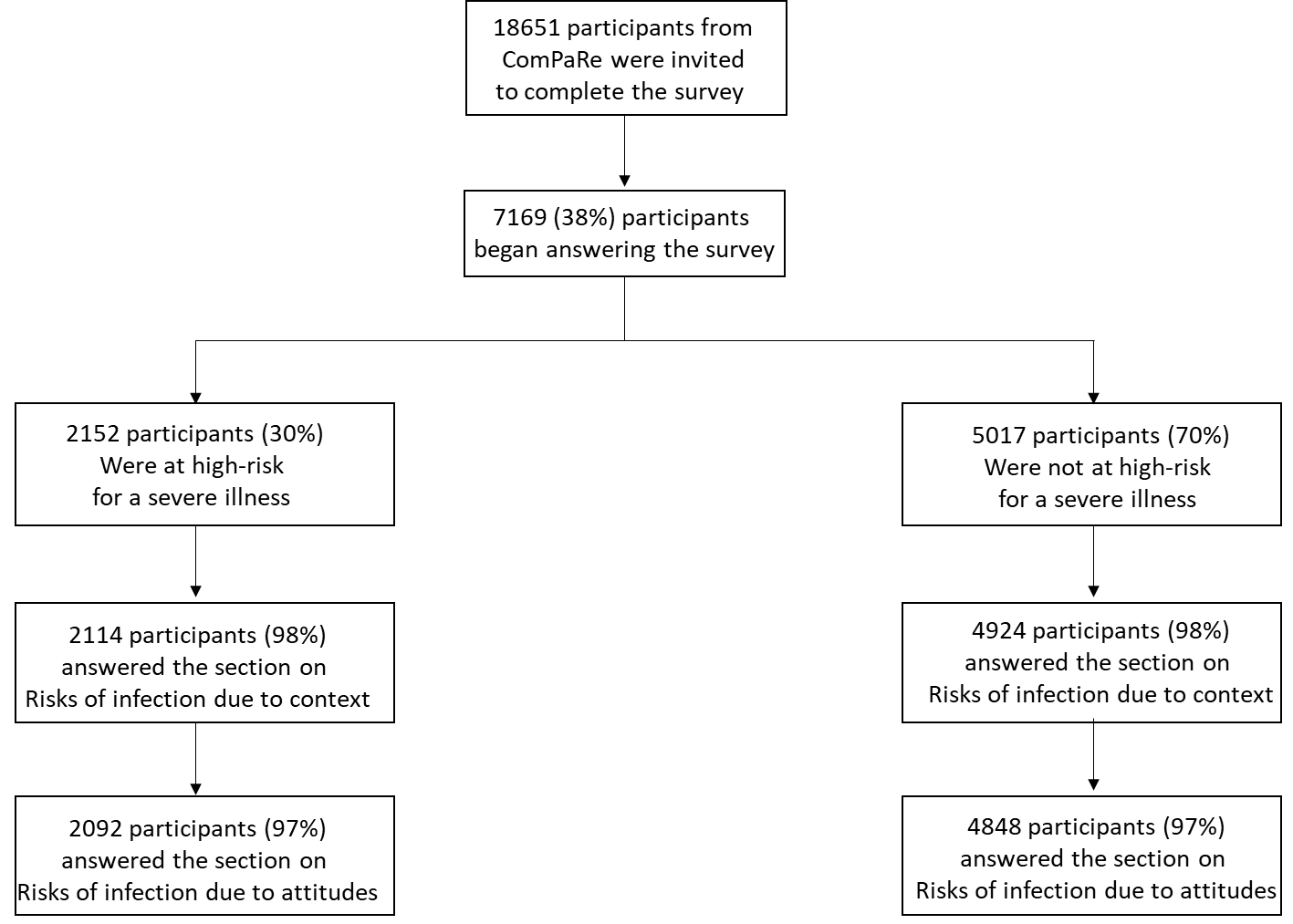
**

Supplement: S1 Fig — (DOCX) [file pone.0237296.s003.docx]
