# Supplementary material for: COVID-19–related perceptions, context and attitudes of adults with chronic conditions: Results from a cross-sectional survey nested in the ComPaRe e-cohort
Source: PLoS One. 2020 Aug 6;15(8):e0237296. doi: 10.1371/journal.pone.0237296 (PMC7410193; doi:10.1371/journal.pone.0237296)
Supplement: S1 Table — (DOCX) [file pone.0237296.s004.docx]

**S1 Table. Demographic characteristics of respondents and non-respondents to the survey**

| **Characteristic** | **Respondents**  **(n=7169)** | **Non-respondents**  **(n=11482)** |
| --- | --- | --- |
| **Age, mean (SD) – yr** | 46.1 (14.7) | 40.6 (13.0) |
| **Female sex – no (%)** | 5616 (78.3) | 9671 (84.2) |
| **Educational level – no (%)**  Low  Middle school or equivalent  High school or equivalent  Associate’s degree  Higher education | 386 (5.4)  1164 (16.2)  533 (7.4)  1510 (21.1)  3576 (49.9) | 692 (6.0)  2112 (18.4)  1028 (9.0)  2361 (20.6)  5282 (46.0) |
| **Multimorbid – no (%)** | 3684 (51.4) | 3917 (34.1) |
| **Number of diseases, mean (SD)** | 2.3 (2.2) | 1.69 (1.42) |
| **Self-reported conditions**^2^ **– no (%)**  High blood pressure  Diabetes  Stroke or cardiac ischemic disease  Heart failure (other than ischemic diseases)  Asthma  COPD  Thyroid disease  Chronic kidney failure  Cancer  Osteoarthritis  Inflammatory rheumatic diseases | 834 (11.6)  506 (7.1)  70 (2.9)  79 (1.1)  448 (6.2)  124 (1.7)  362 (5.0)  142 (2.0)  373 (5.2)  319 (4.4)  407 (5.7) | 574 (5.0)  491 (4.3)  40 (0.3)  49 (0.4)  541 (4.7)  85 (0.7)  419 (3.6)  90 (0.8)  391 (3.4)  291 (2.5)  404 (3.5) |

COPD, chronic obstructive pulmonary disease
