# Supplementary material for: COVID-19–related perceptions, context and attitudes of adults with chronic conditions: Results from a cross-sectional survey nested in the ComPaRe e-cohort
Source: PLoS One. 2020 Aug 6;15(8):e0237296. doi: 10.1371/journal.pone.0237296 (PMC7410193; doi:10.1371/journal.pone.0237296)
Supplement: Attachment — Submitted filename: Response.docx [file pone.0237296.s005.docx]

Dear Editor,

Thank you very much for giving us the possibility to answer the main concerns raised by the two reviewers of our manuscript entitled “COVID-19–related perceptions, context and attitudes of adults with chronic conditions: results from a cross-sectional survey nested in the ComPaRe e-cohort” (PONE-D-20-14011).

**Journal Requirements:**

**1. Please ensure that your manuscript meets PLOS ONE's style requirements, including those for file naming.**

The article now meets PLOS ONE’s style requirements.

**2. Please include additional information regarding the survey or questionnaire used in the study and ensure that you have provided sufficient details that others could replicate the analyses. For instance, if you developed a questionnaire as part of this study and it is not under a copyright more restrictive than CC-BY, please include a copy, in both the original language and English, as Supporting Information."**

The survey is now available in appendix in both French and English (S1 and S2).

**3. Please state whether you validated the questionnaire prior to testing on study participants. Please provide details regarding the validation group within the methods section.**

The questionnaire was face-validated with several physicians and researchers with expertise in questionnaire development. This is now specified in the methods section

**4. We note that you have indicated that data from this study are available upon request. PLOS only allows data to be available upon request if there are legal or ethical restrictions on sharing data publicly.**

Data from this study are from the ComPaRe e-cohort. Patients participate in the cohort to accelerate research on their chronic conditions. As specified in their consent on inclusion in the cohort, all data collected may be shared and reused by academic researchers after acceptance of their study protocol by the scientific committee of the ComPaRe e-cohort. The procedure is described in detail on the ComPaRe e-cohort website ([www.compare.aphp.fr](http://www.compare.aphp.fr)) and was validated by the ethics committee and the French regulatory body on data privacy (Commission nationale de l'informatique et des liberté).

**a) If there are ethical or legal restrictions on sharing a de-identified data set, please explain them in detail (e.g., data contain potentially sensitive information, data are owned by a third-party organization, etc.) and who has imposed them (e.g., an ethics committee). Please also provide contact information for a data access committee, ethics committee, or other institutional body to which data requests may be sent.**

Data from this study are available for all researchers under the rules of the ComPaRe e-cohort. The procedure is described in detail on the ComPaRe e-cohort website ([www.compare.aphp.fr](http://www.compare.aphp.fr)) and was validated by the ethics committee and the French regulatory body on data privacy (Commission nationale de l'informatique et des liberté).

**5. Thank you for stating the following financial disclosure: The funders had no role in study design, data collection and analysis, decision to publish, or preparation of the manuscript.**

We added financial disclosures at the end of the manuscript.

**At this time, please address the following queries:**

a. **Please clarify the sources of funding (financial or material support) for your study. List the grants or organizations that supported your study, including funding received from your institution.**

Done. No specific grant was obtained for this study.

b. **State what role the funders took in the study. If the funders had no role in your study, please state: “The funders had no role in study design, data collection and analysis, decision to publish, or preparation of the manuscript.”**

Done. We stated that The funders had no role in study design, data collection and analysis, decision to publish, or preparation of the manuscript.”

c. **If any authors received a salary from any of your funders, please state which authors and which funders.**

Done. No salary was obtained by funders.

d. **If you did not receive any funding for this study, please state: “The authors received no specific funding for this work.”**

Done. We added a sentence: The authors received no specific funding for this work.

Please include your amended statements within your cover letter; we will change the online submission form on your behalf.

**6. Please include captions for your Supporting Information files at the end of your manuscript, and update any in-text citations to match accordingly.**

Done

**Reviewer #1: This is peer-review of paper by Viet-Thi Tran et al, which determined Potential risk of COVID-19 in adults with chronic conditions because of their perceptions and attitudes in France. They found that those with chronic medical illnesses have skewed views about their likelihood about severe disease with COVID-19.**
**The strengths of this study are the large sample size.**

**Major Comments:
1. The survey design was omitted for us as evaluators, who designed it? How? Is it validated? Elaborate more to this. It should be presented in the appendix.**

We apologize for the imprecision. The survey was designed by VTT and PR from the literature and their own expertise. It was then face-validated by two other researchers with expertise in questionnaire development before dissemination. The questionnaire was not tested with patients, but first respondents provided comments in a dedicated open-ended question at the end of the questionnaire, leading to minor reformulations.

We modified the methods section and now provide the questionnaire in appendix (**Appendix 1 and 2**).

*In addition, participants answered a dedicated survey designed by VTT and PR by use of the literature and their own expertise 12. It was then face-validated by two other researchers (IP and CR) with expertise in questionnaire development before dissemination. The questionnaire was not tested with patients; however, the first respondents provided comments in a dedicated open-ended question at the end of the questionnaire, which led to minor reformulations.*

**2. The response rate was very low, what was the reason? How did you distribute it?**

The survey was mailed to participants of the ComPaRe e-cohort. The response rate was relatively low because in the context of the pandemic, we aimed for a quick data collection with a limited time for completion (10 days). In addition, the invitation and reminders involved only e-mails. Therefore, participants having full mailboxes or who changed their e-mail may have led to the lower response rate. Finally, as shown in the Supplementary table 4 (2 in the original version of the paper), non-respondents were younger and less multimorbid than respondents, which could indicate that these populations did not perceive COVID-19 as a risk and did not feel concerned by the study.

We would also like to highlight that such response rate was expected and in line with results from the literature, especially among patients with chronic conditions (Kongsved, JMIR, 2007; Schiøtz, Chron Illn, 2012). Furthermore, recent surveys of COVID-19 in the literature are likely not exceptions, yet most of these studies did not report response rates, so we cannot compare figures (Geldsetzer, JMIR, 2020; Clements, JMIR Public Health Surveill 2020, McFadden, Plos ONE 2020).

We now highlight these points in the discussion

*Third, the response rate was relatively low (38%) owing to the short duration of data collection (10 days) and the sole use of e-mails for invitation and reminders. Yet, such response rate is consistent with the literature of online surveys for the general public.*

**3. The analysis was mainly descriptive with no interesting associations came out from this survey. Make it more interesting for the readers and researchers.**

We rephrased multiple parts of the results section to emphasize the importance of findings. In addition, we added two analyses aimed at exploring the relationship between participants’ characteristics and 1) their perception of their risk of severe COVID-19 and 2) their attitudes to prevent infection with occasional contacts with asymptomatic people. To avoid redundancy, results from the logistic regressions replace the previous appendix 3 and 4.

We detailed these analyses in the Methods section:

*In addition, we fitted two logistic regression analyses aimed at exploring the relationship between participants’ characteristics and 1) their perception of their risk of severe COVID-19 and 2) their attitudes on preventing infection with occasional contacts with asymptomatic people. Variables included in the model were sex, age (as a continuous variable), household with > 1 person (including the patient), low educational level, treatment considered at risk according to the French High Council for Public Health, BMI ≥40 kg/m2, high blood pressure, diabetes (under insulin treatment or not), history of stroke or cardiac ischemic disease, heart failure (any New York Heart Association stage), asthma, chronic obstructive pulmonary disease, thyroid disease, chronic kidney failure (under dialysis or not), cancer (under treatment or not) and osteoarthritis. Analyses were performed on complete cases only.*

And in the results section:

**Table 2: Association between participant characteristics and their perceived risk of severe COVID-19.** Results of logistic regression analysis of complete cases, accounting for weights obtained after calibration on margins for sex, age categories and educational level by using data from a national census describing the French population self-reporting at least one chronic condition.

| **Characteristic** | **Odds ratio**  **(95% confidence interval)** |
| --- | --- |
| **Female sex** | 1.33 (1.03–1.7)* |
| **Age** | 1 (1–1.01) |
| **Household > 1 person** | 1.11 (0.82–1.5) |
| **Low educational level** | 1.1 (0.76–1.59) |
| **High-risk treatments** | 13.93 (6.09–31.83)* |
| **BMI ≥ 40 kg/m^2^** | 1.75 (0.8–3.81) |
| **High blood pressure** | 1.12 (0.75–1.68) |
| **Diabetes** | 3.03 (1.84–4.98)* |
| **Stroke or cardiac ischemic disease** | 9.01 (2.22–36.55)* |
| **Heart failure** | 2.3 (0.67–8.43) |
| **Asthma** | 4.58 (2.2–9.56)* |
| **Chronic obstructive pulmonary disease** | 6.27 (2.12–18.51)* |
| **Thyroid disease** | 1.04 (0.55–1.96) |
| **Chronic kidney disease** | 2.6 (1–6.78) |
| **Cancer** | 1.91 (0.98–3.71) |
| **Osteoarthritis** | 1.01 (0.61–1.67) |

* P < 0.05

**Table 3: Association between participant characteristics and the use of face masks for occasional contacts with asymptomatic people (or the refusal to see these people).** Results of logistic regression analysis of complete cases, accounting for weights obtained after calibration on margins for sex, age categories and educational level by using data from a national census describing the French population self-reporting at least one chronic condition.

| **Characteristic** | **Odds ratio**  **(95% confidence interval)** |
| --- | --- |
| **Female sex** | 1.21 (0.95–1.55) |
| **Age** | 1 (0.99–1.01) |
| **Household > 1 person** | 1.21 (0.94–1.57) |
| **Low educational level** | 0.96 (0.68–1.36) |
| **High-risk treatments** | 1.25 (0.83–1.88) |
| **BMI ≥ 40 kg/m2** | 1.4 (0.79 ─2.48) |
| **High blood pressure** | 0.75 (0.53–1.06) |
| **Diabetes** | 1.2 (0.82–1.76) |
| **Stroke or cardiac ischemic disease** | 1.24 (0.53–2.89) |
| **Heart failure** | 2.48 (0.96–6.45) |
| **Asthma** | 1 (0.69–1.45) |
| **Chronic obstructive pulmonary disease** | 1.01 (0.47–2.18) |
| **Thyroid disease** | 1.25 (0.79–1.99) |
| **Chronic kidney disease** | 0.87 (0.41–1.85) |
| **Cancer** | 0.73 (0.47–1.14) |
| **Osteoarthritis** | 1.39 (0.88–2.18) |
| **Feeling at risk øf severe COVID-19** | 1.93 (1.53–2.43)* |

* P < 0.05

**4. The definition of the high risk of severe illness is not clear and misleading.**

We apologize for the confusion. The definition of high risk of severe illness was from the French High Council for Public Health available at <https://www.hcsp.fr/explore.cgi/avisrapportsdomaine?clefr=775>.

We rewrote and moved the section completely and added a box highlighting the different items included in the definition.

**Definition of high risk according to the literature:**

- age ≥ 70 years’ old
- history of cardiovascular disease: complicated hypertension, history of stroke or coronary artery disease, heart surgery, heart failure (New York Heart Association class III or IV);
- insulin-dependent diabetes or diabetic microangiopathy or macroangiopathy
- chronic respiratory disease likely to result in decompensation during a viral infection
- chronic renal failure on dialysis
- cancer under treatment

**Despite the lack of data in the literature, the following patients are also considered at high risk based on available data on other respiratory infections:**

- congenital or acquired immunosuppression due to drug therapy (cancer chemotherapy, immunosuppressive therapy, biotherapy and/or immunosuppressive dose corticosteroid therapy), uncontrolled HIV infection or CD4 count <200/mm3, solid organ or hematopoietic stem cell transplant, or blood cancer under treatment
- cirrhosis at least stage B of the Child-Pugh classification
- morbid obesity (body mass index > 40 kg/m^2^) by analogy with influenza A (H1N1)09
- third trimester of pregnancy

**5. Why did you divide Perception of risk of severe illness with COVID-19 to four categories? Explain further and make a valid point.**

We believe that our methods section was confusing and we completely rewrote it.

Participants could “feel” at risk of severe COVID-19 and/or be at risk (according to French recommendations) for severe COVID-19. For example, a patient with only osteoarthritis is not at high risk of severe COVID-19 according to recommendations but may “feel” at risk. Therefore, this defined 4 categories, which are presented in Figure 1.

**6. Your survey was looking for perception of people but attitude of people was presented and analysed, please be specific about your objectives and don’t confuse the readers.**

We apologize and reformulated the objectives of the study in the title, abstract and objectives of the study.

Title: *COVID-19–related perceptions, context and attitudes of adults with chronic conditions: results from a cross-sectional survey nested in the ComPaRe e-cohort*

Background of abstract: *To avoid a surge of demand on the healthcare system due to the COVID-19 pandemic, we must reduce transmission to individuals with chronic conditions who are at risk of severe COVID-19. We investigated the perceptions, context or attitudes toward COVID-19 of individuals with chronic conditions to better understand their potential risk of infection.*

Objectives: *In this study, we aimed to evaluate the perceptions, context and attitudes toward COVID-19 of individuals with chronic conditions in order to better understand their potential risk of infection.*

**7. Very limited discussion was presented; no scientific discussion has been done which very important papers were not discussed concerning prevalence, severity and mortality of chronic respiratory conditions, cardiac, renal, and other comorbidities.**

We considerably expanded the discussion
