# Supplementary material for: COVID-19–related perceptions, context and attitudes of adults with chronic conditions: Results from a cross-sectional survey nested in the ComPaRe e-cohort
Source: PLoS One. 2020 Aug 6;15(8):e0237296. doi: 10.1371/journal.pone.0237296 (PMC7410193; doi:10.1371/journal.pone.0237296)
Supplement: Attachment — Submitted filename: Response3.docx [file pone.0237296.s006.docx]

Dear Editor,

Thank you for giving us the possibility to answer the main concerns raised by the two reviewers of our manuscript entitled “COVID-19–related perceptions, context and attitudes of adults with chronic conditions: results from a cross-sectional survey nested in the ComPaRe e-cohort” (PONE-D-20-14011R1).

Reviewers mainly asked for changes in the discussion to detail the link between COVID-19 infection and chronic conditions (e.g. discussing the prevalence, severity and mortality of chronic respiratory conditions, cardiac, renal, and other comorbidities in France). These are, of course, topics of critical importance but we are not sure that they are within the scope of the paper, which was to explore patients’ perceptions and attitudes toward the risk of COVID-19. As requested by the reviewers, we added more details in the discussion to provide a basis for reflection, but we believe that expanding this section too much may lead readers astray.

**Reviewer #1: Thank you for the authors for addressing all the comments except ONE, which is number (7. A very limited discussion was presented; no scientific discussion has been done which very important papers were not discussed concerning prevalence, severity and mortality of chronic respiratory conditions, cardiac, renal, and other comorbidities). They need to cite systematic reviews and meta-analysis about the above comorbidities and link that with the current prevalence and severity reported in France.**

We added some details to discuss the importance of understanding the perceptions of patients with chronic conditions toward COVID-19.

*Our results are important given the cumulative amount of evidence showing that patients with chronic conditions, about 20 million individuals in France, are at increased risk of severe COVID and death. In a small case series conducted at the beginning of the epidemic in China, among 102 patients hospitalized for COVID-19, those with comorbidities (especially hypertension, diabetes, cardiovascular and respiratory diseases) were more likely to be hospitalized in Intensive care units. Similar findings were observed in Europe. In a large case series of 4000 patients hospitalized in ICUs in Italy, the highest risk of death was for patients with chronic obstructive pulmonary disease (adjusted HR [aHR] 1.68, 95% CI 1.28-2.19) and type 2 diabetes (aHR 1.18, 95% CI 1.01-1.39). Reasons underlying these findings are still unclear, with hypotheses related to meta-inflammation or use of angiotensin-converting enzyme inhibitors (ACEIs)/angiotensin receptor blockers (ARBs) in these populations, despite recent controversial findings about this latter point.*

**The other important point here is about Attitude, I cannot see anything about smoking habits and the risk of getting an infection with those vulnerable group of patients. A recent meta-analysis published by Plos One highlighted the prevalence and severity associated with former, never and current smokers and COVID-19. Please discuss this in your discussion and make a comparison with your results as this definitely ATTITUDE.**

Our objective was to assess patients’ perceptions regarding COVID-19 and their attitudes regarding protection measures. We did not collect whether patients stopped smoking because of the fear of severe COVID. However, we added details about patients’ current smoking status and their perception of risk for severe COVID-19.

Especially, we now describe in table 1 patients’ smoking status:

| **Smoking status**  Never smoker  Former smoker  Current smoker (occasional)  Current smoker (frequent)  Missing | 2319 (46.3)  1671 (33.3)  290 (5.8)  732 (14.6)  2 (0.03) | 2554 (35.6)  3371 (47)  309 (4.3)  926 (12.9)  9 (0.1) |
| --- | --- | --- |

Furthermore, we added patients’ smoking status in the two models, which did not change the results.

**Table 2: Association between participant characteristics and their perceived risk of severe COVID-19.** Results of logistic regression analysis of complete cases, accounting for weights obtained after calibration on margins for sex, age categories and educational level by using data from a national census describing the French population self-reporting at least one chronic condition.

| **Characteristic** | **Odds ratio**  **(95% confidence interval)** |
| --- | --- |
| **Female sex** | 1.33 (1.04–1.71)* |
| **Age** | 1.01 (1–1.02)* |
| **Current smoker** | 1.21 (0.9–1.62) |
| **Household > 1 person** | 1.12 (0.83–1.52) |
| **Low educational level** | 1.1 (0.76–1.59) |
| **High risk treatments** | 14.08 ( 6.16–32.18)* |
| **BMI ≥ 40 kg/m2** | 1.77 (0.81–3.87) |
| **High blood pressure** | 1.13 (0.75–1.69) |
| **Diabetes** | 3.02 (1.83–4.98)* |
| **Stroke or cardiac ischemic disease** | 9.16 (2.26–37.19)* |
| **Heart failure** | 2.4 (0.67–8.63) |
| **Asthma** | 4.64 (2.22–9.68)* |
| **COPD** | 6.32 (2.14–18.73)* |
| **Thyroid disease** | 1.05 (0.55–1.97) |
| **Chronic kidney disease** | 2.64 (1.01–6.89)* |
| **Cancer** | 1.93 (0.99–3.73) |
| **Osteoarthritis** | 0.98 (0.59–1.62) |

* P < 0.05

BMI, body mass index; COPD, chronic obstructive pulmonary disease

**Table 3: Association between participant characteristics and the use of face masks for occasional contacts with asymptomatic people (or the refusal to see these people).** Results of logistic regression analysis of complete cases, accounting for weights obtained after calibration on margins for sex, age categories and educational level by using data from a national census describing the French population self-reporting at least one chronic condition.

| **Characteristic** | **Odds ratio**  **(95% confidence interval)** |
| --- | --- |
| **Female sex** | 1.18 (0.92–1.5) |
| **Age** | 1 (0.99–1.01) |
| **Current smoker** | 0.94 (0.69–1.28) |
| **Household > 1 person** | 1.2 (0.93–1.56) |
| **Low educational level** | 0.95 (0.67–1.34) |
| **High-risk treatments** | 1 (0.65–1.53) |
| **BMI ≥ 40 kg/m2** | 1.31 (0.73–2.35) |
| **High blood pressure** | 0.73 (0.52–1.03) |
| **Diabetes** | 1.06 (0.72–1.55) |
| **Stroke or cardiac ischemic disease** | 1.04 (0.44–2.48) |
| **Heart failure** | 2.36 (0.9–6.23) |
| **Asthma** | 0.85 (0.59–1.23) |
| **COPD** | 0.89 (0.42–1.87) |
| **Thyroid disease** | 1.25 (0.79–1.97) |
| **Chronic kidney disease** | 0.83 (0.4–1.73) |
| **Cancer** | 0.66 (0.43–1.03) |
| **Osteoarthritis** | 1.42 (0.91–2.23) |
| **Feeling at risk of severe COVID-19** | 1.93 (1.53–2.44) |

* P < 0.05

We discussed the fact that patients with BMI ≥ 40 kg/m2 or who smoked did not feel at risk and did not take specific measures to protect themselves despite these two factors being associated with risk of severe complications and mortality from COVID-19.

*Especially, our findings highlight that patients with a BMI ≥ 40 kg/m² or who smoked did not feel at risk nor took extra precautions when in contact with other people despite these two factors being associated with risk of severe complications and mortality from COVID-19*

**Reviewer #2: The authors studied the perceptions, context and attitudes of individuals with chronic conditions during the COVID-19 pandemic to clarify their potential risk of infection, the result was that 63% patients felt at risk because of severe illness, about 23.7% were at risk of infection, at the end they concluded that individuals with chronic conditions have distorted perceptions of their risk of severe illness with COVID-19.So I will give some comments as followings.
In this study, the investigation time period was selected from 3.23-4.2. I want to know the reason for choosing this study time? Why not choosing a longer period of time or another time period?**

The study was conducted at the peak of the epidemic in France and our results were intended to provide a quick insight into the perceptions and attitudes of chronic patients toward the risk for severe infection.

We now report in the settings section, the number of confirmed cases at the time of the study.

*Data from this study were collected between March 23 and April 2, 2020 at the peak of the French epidemic. During that time, 27 475 new cases of COVID-19 were confirmed, with a total of 56 261 cases on April 2, 2020. This time period includes the maximum number of daily cases in France (April 1, 2020).*

Because patients are recruited from an existing cohort, so a longer response period would have, at best, increased the survey response rate. We already describe the difference between respondents and non-respondents and discuss the response rate in comparison to other surveys conducted at the same time.

**In this study, how to define a serious disease state? What types of diseases are included?**

Any participant with a chronic condition can participate in ComPaRe. Patients self-report their chronic conditions by using a list of 217 diseases, inspired from the International Classification of Primary Care-Version 2.

Details on the ComPaRe e-cohort have been published elsewhere (Tran VT, J Clin Epidemiol 2020). The full protocol of the ComPaRe cohort is available on the cohort website www.compare.aphp.fr.

**Whether different disease types will affect the conclusions of this investigation**

Associations identified in this study were independent of the presence of several conditions (high blood pressure, diabetes, stroke, ischemic heart failure, asthma, COPD, thyroid disease, chronic kidney disease, cancer and osteoarthritis). Indeed, these diseases were included as predictors in the non-parsimonious logistic regression analysis exploring the association between patient characteristics, their perception of their risk for severe infection (Table 2) and their use of face masks for occasional contacts with asymptomatic people (Table 3).

**In the face of the COVID-19 epidemic, what are the current policy measures adopted by the French government?**

At the time of the study, France had been under lockdown (movement restrictions and closure of non-essential businesses) and people with chronic conditions were encouraged to stay at home. Yet public communication for people with chronic conditions was imprecise. In particular, there was no consensus on the use of face-masks.

*Since March 17, France had been under lockdown (movement restrictions and closure of non-essential businesses), and people with chronic conditions were encouraged to stay at home. During this time, knowledge of COVID-19 was still limited and information for the public was imprecise. For example, information available on the website of the French ministry of health referred to “people at risk”, mixing older people and patients with chronic conditions.*

**The following references should be discussed in the revision text.
Cao JL, Hu XR, Tu WJ., & Liu Q. (2020). Clinical Features and Short-term Outcomes of 18 Patients with Corona Virus Disease 2019 in Intensive Care Unit. Intensive Care Medicine, DOI: 10.1007/s00134-020- 05987-7.
Cao JL, Tu WJ, Hu XR, & Liu Q. (2020). Clinical Features and Short-term Outcomes of 102 Patients with Corona Virus Disease 2019 in Wuhan,China. Clinical Infectious Diseases,DOI: 10.1093/cid/ciaa243/ 5814897.**

We discuss the aforementioned references in the added paragraph in the discussion.

*Our results are important given the cumulative amount of evidence showing that patients with chronic conditions, about 20 million individuals in France, are at increased risk of severe COVID and death. In a small case series conducted at the beginning of the epidemic in China, among 102 patients hospitalized for COVID-19, those with comorbidities (especially hypertension, diabetes, cardiovascular and respiratory diseases) were more likely to be hospitalized in Intensive care units. Similar findings were observed in Europe. In a large case series of 4000 patients hospitalized in ICUs in Italy, the highest risk of death was for patients with chronic obstructive pulmonary disease (adjusted HR [aHR] 1.68, 95% CI 1.28-2.19) and type 2 diabetes (aHR 1.18, 95% CI 1.01-1.39). Reasons underlying these findings are still unclear, with hypotheses related to meta-inflammation or use of angiotensin-converting enzyme inhibitors (ACEIs)/angiotensin receptor blockers (ARBs) in these populations, despite recent controversial findings about this latter point.*
